# Resveratrol is a promising agent for colorectal cancer prevention and treatment: focus on molecular mechanisms

**DOI:** 10.1186/s12935-019-0906-y

**Published:** 2019-07-15

**Authors:** Mohadese Honari, Rana Shafabakhsh, Russel J. Reiter, Hamed Mirzaei, Zatollah Asemi

**Affiliations:** 1 Research Center for Biochemistry and Nutrition in Metabolic Diseases, Kashan University of Medical Sciences, Kashan, Islamic Republic of Iran; 2 Department of Cellular and Structural Biology, University of Texas Health Science, Center, San Antonio, TX USA

**Keywords:** Colorectal cancer, Resveratrol, Anti-inflammatory, Anti-oncotic

## Abstract

Colorectal cancer (CRC) is the third most common cancer and one of the main causes of cancer death entire the world. Environmental, dietary, and lifestyle factors including red meat consumption, cigarette smoking, alcohol intake and family history are the most important risk factors of CRC. Multiple pathways including inflammation, oxidative stress, and apoptosis are involved in its incidence and progression. Resveratrol, a polyphenolic compound, has different pharmacologic functions including anti-inflammation, cancer prevention, lipid-lowering effect, and hypoglycemic effect. Many studies have proved that resveratrol might also represent a chemo preventive effect on CRC. Thus, the aim of the current review is to depict the role of resveratrol in treatment of CRC in a molecular manner.

## Background

Colorectal cancer (CRC) is the third most common cancer global [1]. Hepatic metastasis is the leading cause of mortality in this cancer [2]. CRC has caused about 0.69 million deaths, and its mortality rate is still growing up. The international Cancer Statistic indicated that there are 1.36 million new CRC cases annually entire the world [3]. In Western countries, particularly in the United States, there is a recent decrease in CRC incidence rate that is due to increased screening such as colonoscopy and fecal occult blood test [4]. Enhanced screening contributes to early detection and treatment of precancerous polyps [5, 6] as in high-income countries, the disease prevalence has been stable because of increased screening [7]. CRC treatment still poses a clinical problem while 5-year survival rates are about 65% which depends on tumor situation, stage of tumor detection and therapy [8]. Secular trend studies suggested that lifestyle, dietary, and environmental factors including red meat intake, cigarette smoking, and alcohol intake are the most important risk factors of CRC [5, 9]. In addition, several studies have shown that a family history of CRC elevated the disease risk. Based on the previous observations, present guidelines propose that people with a family history of this cancer should be screened for CRC earlier than those without one [10]. Recognition of new therapeutic strategies may enhance the survival rate of patients with CRC [11].

Along to various therapies, utilization of natural compounds has been appeared as new horizon in the treatment of a variety of diseases such as cancer [12–14]. In this regards, several in vitro and in vivo investigations have shown that phytochemicals act potential antioxidant, anti-inflammatory and anti-carcinogenic roles by modulating certain signaling pathways and molecular biomarkers to stop the incidence and progression of CRC [15]. Resveratrol (trans-3, 4′, 5-trihydroxystilbene) which derivates from the stilbene family of phenolic compounds, exists in berries, pines and nuts, and particularly in red grapes skin [16]. This compound has different pharmacological functions including anti-inflammation, cancer inhibition, lipid-lowering effect, and hypoglycemic effect. Resveratrol inhibits lipid peroxidation and aggregation of thrombocytes thus plays as an anti-oxidant, anti-inflammatory and vasodilation [17, 18]. Recently, it was found that resveratrol instigates autophagy in cancer cells [19]. Resveratrol has been exhibited to raise the release of various pro-inflammatory cytokines from immune cells, which finally promotes cytotoxicity against cancer [20]. This safe and multi-targeted natural agent has been connected with suppression of survival and invasion of cancer cells [21].

Many studies have proved that resveratrol might also represent a chemo preventive effect on CRC. A recent in vitro study demonstrated that resveratrol in combination with 5-fluorouracil (5-FU), a chemotherapeutic drug, increased the effects of 5-FU via its effects as an anti-metastasis drug on CRC. It was prominent that 5-FU resistant cells were more susceptible to resveratrol illustrating a potential therapeutic strategy for 5-FU resistant CRC [21]. Previous studies also reported that resveratrol increases the anti-cancer activities of the chemotherapeutic agent, oxaliplatin, in cell culture model of CRC. Resveratrol can synergistically increase the effects of oxaliplatin in tumor cell growth suppression [22].

Given that resveratrol is not considered as primary therapy in the treatment of cancer [23]. Multiple lines evidence indicated that resveratrol could be employed as the secondary line of therapy and primary line cancer therapies alongside resveratrol show significant results [23, 24]. In this regards, a wide ranges of studies confirmed that resveratrol with radiotherapy approaches or chemotherapies could be provided a good therapeutic regimen in the treatment of cancer patients [24, 25].

Metabolic kinetic studies have exhibited that resveratrol have high clearance rate in circulation and low bioavailability [26–28], thus its application for CRC inhibition has been highly disputed. Some studies have recommended that the possible reason for low amount of resveratrol in the circulation is that resveratrol induces the phase II metabolic enzymes UGT and sulfotransferase as soon as possible to generate resveratrol glucuronides and resveratrol sulfates [29, 30]. A recent clinical trial has clarified that the level of three resveratrol sulfate conjugates was increased in patients with oral uptake of 0.5–1.0 g resveratrol each day and the progression of tumor cells was remarkably inhibited in them, proposing that daily oral administration of 0.5–1.0 g resveratrol might act a potential role in CRC inhibition [31]. Thus, the aim of the current review is to depict the role of resveratrol in treatment of CRC in a molecular manner.

## Resveratrol as an anti-inflammatory agent in CRC

**Fig. 1:**
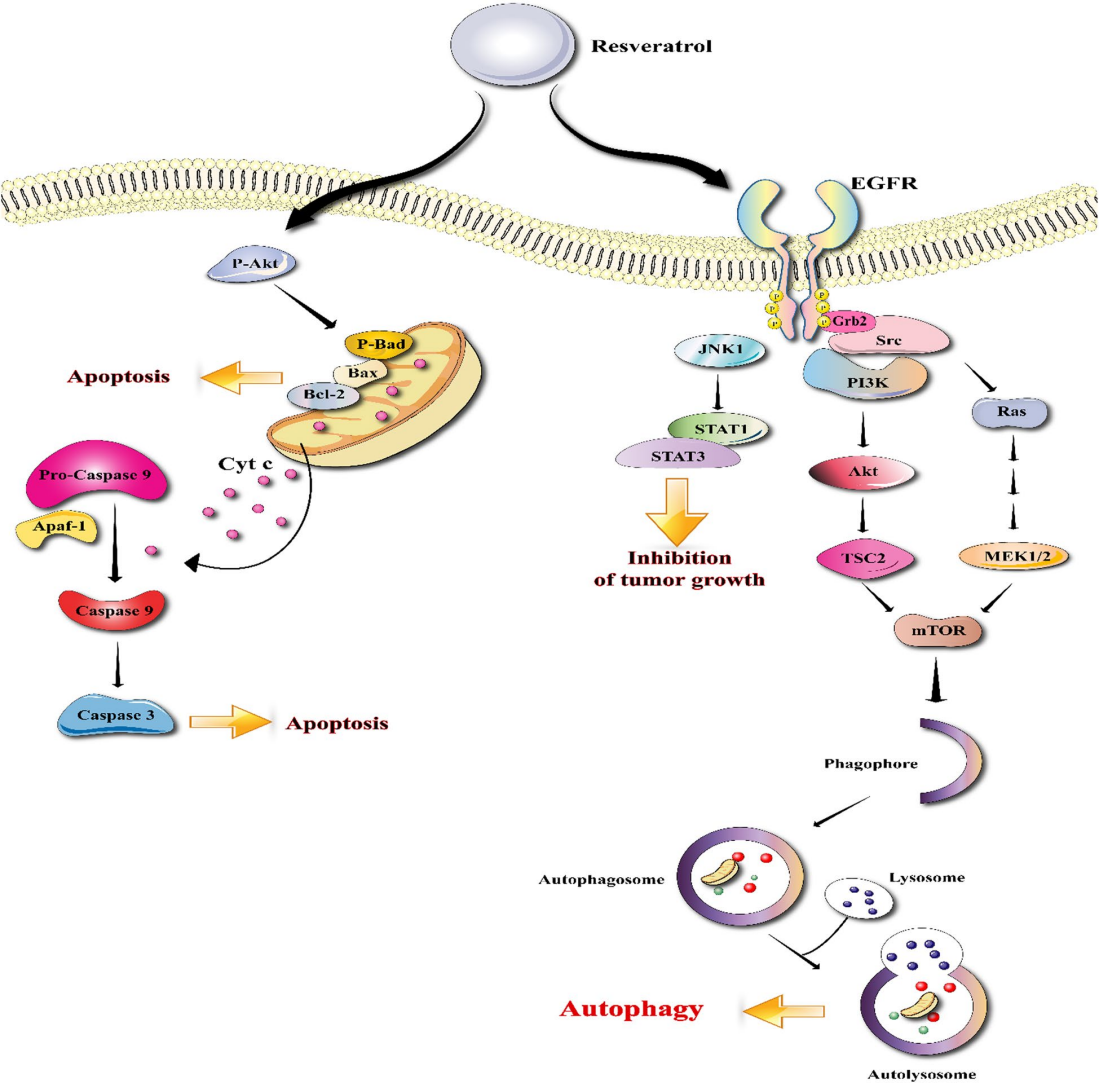
Schematic representation in targeting different signaling pathways using resveratrol as a novel therapeutic strategy in the treatment of colorectal cancer

Chronic inflammation is one of the main mechanisms which involved in colon cancer. Therefore, Anti-inflammatory compounds may be beneficial in treatment of CRC. Cytokines are quickly released by damaged tissues and are stimulators of inflammatory response [32, 33]. Very recently studies have indicated that exposure of intestinal cells to cytokines can mobilize inflammatory pathways such as MAPKs, JAK-STAT and NF-kB cascades, raise the expression of pro-inflammatory enzymes, induce the generation of pro-inflammatory mediators and also production of reactive oxygen species (ROS) [34, 35]. Resveratrol decreases pro-inflammatory mediators, such as TNF-α and IL-1β, pro-inflammatory enzymes such as iNOS and COX-2 and inflammatory signaling pathways such as NF-kB (Fig. 1) [36].

Cancer cell metastasis and invasion are interconnected processes which involve cell proliferation, cell adhesion, cell migration, and proteolytic degeneration of tissue barriers such as the ECM and basal membrane. Multiple proteolytic enzymes which include matrix metalloproteinase (MMPs; especially MMP-2 and MMP-9) [37, 38] and intracellular adhesion molecule (ICAM; especially ICAM-1), take part in the degeneration of these barriers [39, 40]. Some of studies in pancreatic, breast, lung, and colon cancers have exhibited over-expression of MMPs in virulent tissues compared with near normal tissues [41, 42]. Resveratrol diminished the migration and invasion of lung cancer cells due to suppression of NF-κB activation and MMP-2 and MMP-9 expression [43]. Previous studies have demonstrated that resveratrol works as a multi-tasking factor and has anti-inflammatory and anti-cancer functions in CRC [44, 45]. Recent studies have proved that resveratrol inhibits NF-κB-dependent signaling mechanisms [15]. Various pro-inflammatory mediators that have been demonstrated to change the tumor microenvironment, including members of tumor necrosis factor (TNF)-superfamily, are modulated by NF-kB [9, 10]. TNF-α which is generated in the tumor microenvironment, modulates cancer cells, their surrounding stromal cells and the ECM in multiple cancers and acts as an autocrine and paracrine growth factor [46, 47]. Recent study reported that resveratrol suppressed the invasion and viability of CRC cells induced by TNF-α and TNF-β [8]. Another study reported that resveratrol inhibited TNF-β and TNF-α-enhanced survival in CRC cells by increasing apoptotic factors such as cleaved caspase-3 [48]. This study also showed that resveratrol decreased NF-κB activation, NF-κB-dependent carcinogenic gene products (MMP-9 and CXCR4) as well as EMT-related signaling factors including vimentin, slug, and E-cadherin in CRC cells. There is also some evidence which showed that resveratrol can inhibit MMP-9 and vascular endothelial growth factor (VEGF) signaling pathways. Suppression of MMP-9 and VEGF by resveratrol prevents metastasis and angiogenesis [49, 50]. Nitric oxide (NO) is produced by three isozymes of NO synthase. One of these izosymes is iNOS which is inducible by cytokines and pro-inflammatory mediators and is calcium independent while other ones are calcium/calmodulin dependent and constitutively produced [51]. In human and animal models of CRC, it has been indicated that the activation and expression of iNOS is increased suggesting the significant role of NO in colon tumorigenesis [52]. Several studies have also reported that resveratrol decreased iNOS expression in colon cancer cells however its main mechanism is still unclear [53]. Pterostilbene (trans-3, 5-dimethoxy-4′-hydroxystilbene), a structural analogue of resveratrol, exerts an anti-inflammatory action for inhibition of colon carcinogenesis which inhibits p38 MAPK signaling pathway leading to induction of COX-2 and iNOS [35]. In addition, resveratrol is a profitable, nontoxic supplementation and alternative strategy to decrease colitis and strongly colon cancer related to colitis. Resveratrol remarkably develops inflammation score, decreases the amount of neutrophils in the mesenteric lymph nodes and lamina propria, and regulates CD3(+) T cells that release TNF-α and IFN-γ. P53, a marker of inflammation, is also diminished by resveratrol [54].

## Resveratrol inhibits oxidative stress in CRC

At the moment, oxidative stress has been growing up assumed as a main enhancer to carcinogenesis [55]. Several studies have been concentrated on chemo preventive exert of resveratrol as an antioxidant against aging, cardiovascular diseases and cancer. The antioxidant activity of resveratrol is mainly associated with its ability in inhibition free radical production, lipid peroxidation, and modulation of antioxidant-associated enzymes [56]. One of the important antioxidant enzymes is manganese superoxide dismutase, which converts damaging free radical, superoxide, into molecular oxygen and hydrogen peroxide [49]. Resveratrol suppresses the expression of this enzyme and therefore contributes to ROS inhibition [56]. Resveratrol has already represented suppressor effects on peroxynitrite-mediated proteins and lipids oxidation [57] via up-regulation of SOD [58], catalase (CAT), glutathione peroxidase (GPX) [59] and activation of the Sirt1/AMPK and Nrf2 pathways [60].

Two substantial signaling molecules, the replication factor STAT3 and the protein kinase AKT, have been reported to be over-expressed or activated in most types of human cancers, thus, it is now usually approved that both proteins exhibit reliable goals for new anti-tumor drug design [61, 62]. CRC cell lines were found to represent fundamental activation of survival raising proteins including AKT and STAT3. Resveratrol inhibits AKT and STAT3 proteins that are recognized to have oncogenic ability in CRC [63]. Wallerath et al. [64] demonstrated that resveratrol might quickly enhance NO generation in cultured endothelial cells. At physiologic levels, NO, as an antioxidant, supports the gastrointestinal mucosa from injuries, prevents leukocyte sticking, and protects mucosal blood flow [65, 66].

## Resveratrol decreases CRC cell survival

In carcinomas, it has been discovered that integrin-mediated signaling through focal adhesion provides the cytoskeleton reorganization and supports tumor development, invasion and metastasis [67]. Some studies have demonstrated that accumulation of Focal Adhesion Kinase (FAK) with integrins and cytoskeletal proteins in focal contacts causes elevated cell migration, as well as potential modulation of cell growth and survival [68, 69]. Also, in tumors, FAK has been exhibited to be involved in cell migration, survival, invasion and metastasis, gene expression and tumor stem cell self-renewal [70]. Resveratrol suppresses proliferation and invasion of CRC cells via induction of Sirt1 activity, reduction of NF-κB-mediated inflammatory pathway and inhibition of focal adhesion kinase (FAK) activity, leading to decreased focal adhesion molecules and an elevation in apoptosis [71]. Additionally, resveratrol inhibited invasion and colony forming capacity, cell growth, the expression of β1-Integrin and FAK activation of cells in alginate cancer microenvironment. Resveratrol suppressed NF-kB activation and therefore, inhibited NF-κB-dependent gene end-products which are involved in apoptosis, metastasis and invasion [71].

One subgroup of transforming growth factor (TGF)-β superfamily, bone morphogenetic proteins (BMPs), acts important roles in the regulation of several key phases of embryonic development, growth, differentiation and apoptosis of different cells [72]. Recent evidence indicated that BMPs take part in cancer progression, such as colon cancer [73]. BMP starts its signaling via phosphatidyl inositol 3-kinase (PI3K)/Akt pathway [74, 75]. P13K/Akt signaling acts a serious role in regulating cell survival and apoptosis [76] which has been detected over-activated in many tumors to decrease apoptosis and promote proliferation. The tumor inhibitor phosphatase and tensin homolog (PTEN) down-regulates PI3K/Akt signaling and is often inactivated by mutations in several cancer types. Resveratrol reduces the phosphorylation of Akt1/2/3 significantly in CRC cells [77].

Resveratrol also represented dose-dependent suppression of Wnt signaling pathway, which is one of the important pathways in several serious diseases like cancer. Resveratrol decreases expression of Wnt target genes including cyclin D1 and conducting, and suppresses the development of Wnt-induced cells and Wnt-driven CRC cells [78]. Other studies reported that resveratrol decreases the expression of cyclins D1 and D2, which directly regulate cell cycle progression [79]. These molecules are usually induced throughout malignancy [80] and inhibited by anticancer phytochemicals [81]. Also, cell cycle arresting by resveratrol in a variety types of cancer cell lines is well recorded [82]. In addition, resveratrol suppresses the activities of various enzymes related to DNA replication and cell proliferation [83]; these collective effects could be the acceptable reason for the effects of resveratrol on modulation of the cell proliferation [84].

## Clinical trials: resveratrol and colorectal cancer

Despite all documented anticancer features of resveratrol, the majority of the investigations were carried out in pre-clinical and cell-culture methods. Since there are differences in metabolism profile and genetics of humans, and also because the potential properties of resveratrol in animal models cannot be supposed equal to humans, the physiological impacts of resveratrol were also studied in humans.

The toxicity, metabolism, and pharmacokinetics of resveratrol have been evaluated in cancer patients and healthy subjects [85, 86]. Resveratrol is swiftly metabolized, mostly into sulfate conjugates and glucuronide which are excreted through the urine. Due to the low bioavailability because of its extensive metabolism, high doses of resveratrol (up to 5 g/day) have been used by investigators. Mentioned researches have revealed that this polyphenolic compound seems to be safe and well-tolerated. However, adverse effects such as abdominal pain nausea and diarrhea, were seen in individuals consume more than 1 g resveratrol daily [86]. Thus, clinical trials are studying this dose limitation [86, 87]. The poor bioavailability of resveratrol is an important problem regarding extrapolation of its impacts to humans, and different methods have been developed to increase its bioavailability, such as taking it with different foods, utilizing it in combination with an additional phytochemical piperine, as well as using a nanotechnological formulations, micronized powders, or prodrug approach [88–93]. Table 1 shows the list of clinical trials on resveratrol and various cancers, including CRC.

**Table 1:** Clinical trials studies on resveratrol and cancer

Cancer	Dose of resveratrol	Duration of study	Number of patient	Outcome	Refs
CRC	5 g/day	14 days	6	Increase the cleaved caspase-3 in malignant hepatic tissue.	[88]
	0.5 or 1 g/day	8 days	20	Decrease expression of Ki-67	[31]
	20 or 80 mg/day	2 weeks	8	Inhibit the Wnt pathway	[100]
PC	150 mg or 1000 mg/day	4 months	66	Decrease the serum levels of androgens with no alterations in prostate tumor growth	[95]
	4000 mg/day		14	Safe	[94]
MM	5 g/day	21 days	24	Unacceptable safety profile and minimal efficacy in patients with relapsed/refractory multiple myeloma highlighting the risks of novel drug development in such populations	[97]
BC	5 or 50 mg twice a day	3 months	39	Reduce the methylation of *RASSF*-*1α*	[101]

*CRC* colorectal cancer, *PC* prostate cancer, *MM* multiple myeloma, *BC* breast cancer

An investigation conducted on the pathogenesis of prostate cancer revealed that resveratrol is able to delay cancer recurrence. Approximately 33–50% of patients with prostate cancer experience biochemical disease recurrence after primary therapy. Increasing concentration of prostate-specific antigen (PSA) is the earliest disease recurrence indication. MPX, pulverized muscadine grape skin containing resveratrol, delayed recurrence development by prolongation of the PSA doubling time (PSADT) by 5.3 months. However, these findings were not significant [94]. This investigation is carrying on in phase II clinical trial, so it remains to be observed if MPX is a viable option of therapy. Conflictingly, a second clinical trial implicating resveratrol and prostate cancer certainly concluded that it would not be a viable therapy. Despite pre-clinical data that resveratrol regulates the activity of androgen receptor and decreases androgen generation, Kjaer et al. [95] indicated that, since it had no impact on PSA levels or prostate volume, resveratrol could not treat prostate cancer. Based on what discussed, it seems improbable that resveratrol will demonstrate to be efficient for prostate cancer therapy, but further clinical trials require to be done to confirm this.

There are other researches that show resveratrol as a tenuous therapy, including in certain multiple myeloma (MM) types. Resveratrol was observed to suppress STAT3, AKT, and NF-κB as well as exerts cytotoxic effects in MM cell lines [96]. SRT501 was examined in subjects with refractory or relapsed MM. Despite convincing evidence of pre-clinical studies that resveratrol contributes to the therapy of MM patients, the clinical trial showed that it caused numerous severe adverse effects, the most prominent of which was renal failure [97]. Since SRT501 had no nephrotoxic effects in a phase I trial, and it was documented to be safe in a phase II study for patients with colorectal cancer, this adverse effects seemed to be MM-specific. These findings show that SRT501 and probably any other resveratrol versions could not be a potential therapy for MM.

Likewise, in clinical trials performed in colorectal cancer patients, the findings seem hopeful, but remain indeterminate regarding whether resveratrol could be an appropriate therapy. It has been revealed that resveratrol suppresses tumor growth and triggers apoptosis in human colon cells in vitro, and murine models indicated that resveratrol suppresses colorectal carcinogenesis and inflammation [98, 99]. Thus, two clinical trials aimed to distinguish resveratrol pharmacokinetics in hepatic metastases or colorectal tissue. After a 2-week resveratrol or SRT501 therapy, in the colorectal cancer tissues of patients, the measured parent resveratrol as well as its main metabolites levels were alike to the effective resveratrol levels utilized in preclinical investigations. However, the anticancer function of the metabolites of resveratrol has yet to be validated, hence it is obscure whether this result provides additional vindication for pursuing resveratrol as a potential therapy for colon cancer. Furthermore, caspase-3 levels, an apoptotic biomarker, and Ki-67 levels, a biomarker of proliferation, were slightly influenced in tissue samples [31, 88]. Although it is proven that resveratrol possesses some pharmacological effects, it is obscure if these effects are considerable enough to make it a beneficial therapeutic material for colon cancer treatment.

## Conclusions

CRC is a prevalent cancer and one of the main causes of cancer mortality entire the world. Several factors from genetics to diet are involved in the incidence of this malignancy. Its pathophysiology is heterogeneous which multiple molecules and various signaling pathways including inflammation, oxidative stress, and apoptosis are implicated in its incidence and progression. A number studies have supported the potential effects of resveratrol in CRC treatment. This polyphenol compound represents different properties including antioxidant, anti-inflammatory, apoptosis inducer, and anti-angiogenesis efficacy. Due to these significant effects, resveratrol is suggested as a novel therapeutic agent for cancers. Moreover, some studies reported that consumption of resveratrol in combination with other anti-cancer drugs can increase their effects and also decrease their side effects. Thus, this multi-tasking compound can be a new candidate in CRC treatment however, more human studies are needed.

## Data Availability

The primary data for this study is available from the authors on direct request.

## Abbreviations

CRC: colorectal cancer
PI3K: phosphatidyl inositol 3-kinase

## References

[1] Reijonen P, Osterlund P, Isoniemi H, Arola J, Nordin A (2018). Histologically verified biliary invasion was associated with impaired liver recurrence-free survival in resected colorectal cancer liver metastases. Scand J Surg.

[2] Helling TS, Martin M (2014). Cause of death from liver metastases in colorectal cancer. Ann Surg Oncol.

[3] Gan Y, Li Y, Li T, Shu G, Yin G (2018). CCNA2 acts as a novel biomarker in regulating the growth and apoptosis of colorectal cancer. Cancer Manag Res.

[4] Fajardo AM, Piazza GA (2015). Chemoprevention in gastrointestinal physiology and disease. Anti-inflammatory approaches for colorectal cancer chemoprevention. Am J Physiol Gastrointest Liver Physiol.

[5] Jemal A, Bray F, Center MM, Ferlay J, Ward E, Forman D (2011). Global cancer statistics. CA Cancer J Clin.

[6] Siegel R, Naishadham D, Jemal A (2013). Cancer statistics, 2013. CA Cancer J Clin.

[7] Torre LA, Bray F, Siegel RL, Ferlay J, Lortet-Tieulent J, Jemal A (2015). Global cancer statistics, 2012. CA Cancer J Clin.

[8] Siegel RL, Miller KD, Fedewa SA, Ahnen DJ, Meester RGS, Barzi A (2017). Colorectal cancer statistics, 2017. CA Cancer J Clin.

[9] Aune D, Chan DS, Lau R, Vieira R, Greenwood DC, Kampman E, Norat T (2011). Dietary fibre, whole grains, and risk of colorectal cancer: systematic review and dose-response meta-analysis of prospective studies. BMJ.

[10] Park CH, Kim NH, Park JH, Park DI, Sohn CI, Jung YS (2019). Impact of family history of colorectal cancer on age-specific prevalence of colorectal neoplasia. J Gastroenterol Hepatol.

[11] Kim DU, Kwak B, Kim SW (2019). Phosphodiesterase 4B is an effective therapeutic target in colorectal cancer. Biochem Biophys Res Commun.

[12] Muhammad N, Steele R, Isbell TS, Philips N, Ray RB (2017). Bitter melon extract inhibits breast cancer growth in preclinical model by inducing autophagic cell death. Oncotarget.

[13] Bhattacharya S, Muhammad N, Steele R, Kornbluth J, Ray RB (2017). Bitter melon enhances natural killer-mediated toxicity against head and neck cancer cells. Cancer Prev Res.

[14] Bhattacharya S, Muhammad N, Steele R, Peng G, Ray RB (2016). Immunomodulatory role of bitter melon extract in inhibition of head and neck squamous cell carcinoma growth. Oncotarget.

[15] Yin TF, Wang M, Qing Y, Lin YM, Wu D (2016). Research progress on chemopreventive effects of phytochemicals on colorectal cancer and their mechanisms. World J Gastroenterol.

[16] Rotelli MT, Bocale D, De Fazio M, Ancona P, Scalera I, Memeo R (2015). IN-VITRO evidence for the protective properties of the main components of the Mediterranean diet against colorectal cancer: a systematic review. Surg Oncol.

[17] Pace-Asciak CR, Hahn S, Diamandis EP, Soleas G, Goldberg DM (1995). The red wine phenolics trans-resveratrol and quercetin block human platelet aggregation and eicosanoid synthesis: implications for protection against coronary heart disease. Clin Chim Acta.

[18] Shankar S, Singh G, Srivastava RK (2007). Chemoprevention by resveratrol: molecular mechanisms and therapeutic potential. Front Biosci.

[19] Wang J, Li J, Cao N, Li Z, Han J, Li L (2018). Resveratrol, an activator of SIRT1, induces protective autophagy in non-small-cell lung cancer via inhibiting Akt/mTOR and activating p38-MAPK. Onco Targets Ther.

[20] Trung LQ, An DTT (2018). Is resveratrol a cancer immunomodulatory molecule?. Front Pharmacol.

[21] Buhrmann C, Yazdi M, Popper B, Shayan P, Goel A, Aggarwal BB (2018). Resveratrol chemosensitizes tnf-beta-induced survival of 5-FU-treated colorectal cancer cells. Nutrients.

[22] Kaminski BM, Weigert A, Scherzberg MC, Ley S, Gilbert B, Brecht K (2014). Resveratrol-induced potentiation of the antitumor effects of oxaliplatin is accompanied by an altered cytokine profile of human monocyte-derived macrophages. Apoptosis.

[23] Carter LG, D’Orazio JA, Pearson KJ (2014). Resveratrol and cancer: focus on in vivo evidence. Endocr Relat Cancer.

[24] Jiang Z, Chen K, Cheng L, Yan B, Qian W, Cao J (2017). Resveratrol and cancer treatment: updates. Ann N Y Acad Sci.

[25] Kma L (2013). Synergistic effect of resveratrol and radiotherapy in control of cancers. Asian Pac J Cancer Prev.

[26] Baur JA, Sinclair DA (2006). Therapeutic potential of resveratrol: the in vivo evidence. Nat Rev Drug Discov.

[27] Gescher AJ, Steward WP (2003). Relationship between mechanisms, bioavailibility, and preclinical chemopreventive efficacy of resveratrol: a conundrum. Cancer Epidemiol Biomarkers Prev.

[28] Delmas D, Lin HY (2011). Role of membrane dynamics processes and exogenous molecules in cellular resveratrol uptake: consequences in bioavailability and activities. Mol Nutr Food Res.

[29] Lancon A, Hanet N, Jannin B, Delmas D, Heydel JM, Lizard G (2007). Resveratrol in human hepatoma HepG2 cells: metabolism and inducibility of detoxifying enzymes. Drug Metab Dispos.

[30] Boocock DJ, Faust GE, Patel KR, Schinas AM, Brown VA, Ducharme MP (2007). Phase I dose escalation pharmacokinetic study in healthy volunteers of resveratrol, a potential cancer chemopreventive agent. Cancer Epidemiol Biomarkers Prev.

[31] Patel KR, Brown VA, Jones DJ, Britton RG, Hemingway D, Miller AS (2010). Clinical pharmacology of resveratrol and its metabolites in colorectal cancer patients. Cancer Res.

[32] Feghali CA, Wright TM (1997). Cytokines in acute and chronic inflammation. Front Biosci.

[33] Beck PL, Wallace JL (1997). Cytokines in inflammatory bowel disease. Mediators Inflamm.

[34] Kolios G, Brown Z, Robson RL, Robertson DA, Westwick J (1995). Inducible nitric oxide synthase activity and expression in a human colonic epithelial cell line, HT-29. Brit J Pharmacol.

[35] Paul S, Rimando AM, Lee HJ, Ji Y, Reddy BS, Suh N (2009). Anti-inflammatory action of pterostilbene is mediated through the p38 mitogen-activated protein kinase pathway in colon cancer cells. Cancer Prev Res.

[36] Liang YC, Huang YT, Tsai SH, Lin-Shiau SY, Chen CF, Lin JK (1999). Suppression of inducible cyclooxygenase and inducible nitric oxide synthase by apigenin and related flavonoids in mouse macrophages. Carcinogenesis.

[37] Sternlicht MD, Werb Z (2001). How matrix metalloproteinases regulate cell behavior. Annu Rev Cell Dev Biol.

[38] Jiang MC, Liao CF, Lee PH (2001). Aspirin inhibits matrix metalloproteinase-2 activity, increases E-cadherin production, and inhibits in vitro invasion of tumor cells. Biochem Biophys Res Commun.

[39] Aimes RT, Quigley JP (1995). Matrix metalloproteinase-2 is an interstitial collagenase. Inhibitor-free enzyme catalyzes the cleavage of collagen fibrils and soluble native type I collagen generating the specific 3/4- and 1/4-length fragments. J Biol Chem.

[40] Kleiner DE, Stetler-Stevenson WG (1993). Structural biochemistry and activation of matrix metalloproteases. Curr Opin Cell Biol.

[41] Lochter A, Bissell MJ (1999). An odyssey from breast to bone: multi-step control of mammary metastases and osteolysis by matrix metalloproteinases. APMIS.

[42] Bramhall SR (1997). The matrix metalloproteinases and their inhibitors in pancreatic cancer. From molecular science to a clinical application. Int J Pancreatol.

[43] Liu PL, Tsai JR, Charles AL, Hwang JJ, Chou SH, Ping YH (2010). Resveratrol inhibits human lung adenocarcinoma cell metastasis by suppressing heme oxygenase 1-mediated nuclear factor-kappaB pathway and subsequently downregulating expression of matrix metalloproteinases. Mol Nutr Food Res.

[44] Chan JY, Phoo MS, Clement MV, Pervaiz S, Lee SC (2008). Resveratrol displays converse dose-related effects on 5-fluorouracil-evoked colon cancer cell apoptosis: the roles of caspase-6 and p53. Cancer Biol Ther.

[45] Temraz S, Mukherji D, Shamseddine A (2013). Potential targets for colorectal cancer prevention. Int J Mol Sci.

[46] Balkwill F (2002). Tumor necrosis factor or tumor promoting factor?. Cytokine Growth Factor Rev.

[47] Balkwill F, Mantovani A (2001). Inflammation and cancer: back to Virchow?. Lancet (London, England).

[48] Albini A, Cesana E, Noonan DM (2011). Cancer stem cells and the tumor microenvironment: soloists or choral singers. Curr Pharm Biotechnol.

[49] Boghossian S, Hawash A (2012). Chemoprevention in colorectal cancer—where we stand and what we have learned from twenty year’s experience. Surgeon.

[50] Kimura Y, Sumiyoshi M, Baba K (2008). Antitumor activities of synthetic and natural stilbenes through antiangiogenic action. Cancer Sci.

[51] Cianchi F, Cortesini C, Fantappie O, Messerini L, Schiavone N, Vannacci A (2003). Inducible nitric oxide synthase expression in human colorectal cancer: correlation with tumor angiogenesis. Am J Pathol.

[52] Pandurangan AK, Esa NM (2013). Dietary non-nutritive factors in targeting of regulatory molecules in colorectal cancer: an update. Asian Pac J Cancer Prev.

[53] Panaro MA, Carofiglio V, Acquafredda A, Cavallo P, Cianciulli A (2012). Anti-inflammatory effects of resveratrol occur via inhibition of lipopolysaccharide-induced NF-kappaB activation in Caco-2 and SW480 human colon cancer cells. Br J Nutr.

[54] Cui X, Jin Y, Hofseth AB, Pena E, Habiger J, Chumanevich A (2010). Resveratrol suppresses colitis and colon cancer associated with colitis. Cancer Prev Res.

[55] Sengottuvelan M, Deeptha K, Nalini N (2009). Resveratrol ameliorates DNA damage, prooxidant and antioxidant imbalance in 1,2-dimethylhydrazine induced rat colon carcinogenesis. Chem Biol Interact.

[56] Wang T, Wang G, Zhang Y, Zhang J, Cao W, Chen X (2018). Effect of lentivirus-mediated overexpression or silencing of MnSOD on apoptosis of resveratrol-treated fibroblast-like synoviocytes in rheumatoid arthritis. Eur J Pharmacol.

[57] Olas B, Nowak P, Kolodziejczyk J, Ponczek M, Wachowicz B (2006). Protective effects of resveratrol against oxidative/nitrative modifications of plasma proteins and lipids exposed to peroxynitrite. J Nutr Biochem.

[58] Xia N, Daiber A, Habermeier A, Closs EI, Thum T, Spanier G (2010). Resveratrol reverses endothelial nitric-oxide synthase uncoupling in apolipoprotein E knockout mice. J Pharmacol Exp Ther.

[59] Chiou YS, Tsai ML, Nagabhushanam K, Wang YJ, Wu CH, Ho CT (2011). Pterostilbene is more potent than resveratrol in preventing azoxymethane (AOM)-induced colon tumorigenesis via activation of the NF-E2-related factor 2 (Nrf2)-mediated antioxidant signaling pathway. J Agric Food Chem.

[60] Tamaki N, Cristina Orihuela-Campos R, Inagaki Y, Fukui M, Nagata T, Ito HO (2014). Resveratrol improves oxidative stress and prevents the progression of periodontitis via the activation of the Sirt1/AMPK and the Nrf2/antioxidant defense pathways in a rat periodontitis model. Free Radic Biol Med.

[61] Van Meter TE, Broaddus WC, Cash D, Fillmore H (2006). Cotreatment with a novel phosphoinositide analogue inhibitor and carmustine enhances chemotherapeutic efficacy by attenuating AKT activity in gliomas. Cancer.

[62] Al Zaid Siddiquee K, Turkson J (2008). STAT3 as a target for inducing apoptosis in solid and hematological tumors. Cell Res.

[63] Santandreu FM, Valle A, Oliver J, Roca P (2011). Resveratrol potentiates the cytotoxic oxidative stress induced by chemotherapy in human colon cancer cells. Cell Physiol Biochem.

[64] Wallerath T, Deckert G, Ternes T, Anderson H, Li H, Witte K (2002). Resveratrol, a polyphenolic phytoalexin present in red wine, enhances expression and activity of endothelial nitric oxide synthase. Circulation.

[65] Russell J, Okayama N, Alexander JS, Granger DN, Hsia CJ (1998). Pretreatment with polynitroxyl albumin (PNA) inhibits ischemia-reperfusion induced leukocyte-endothelial cell adhesion. Free Radic Biol Med.

[66] Thom SR, Bhopale VM, Milovanova TN, Yang M, Bogush M, Buerk DG (2013). Nitric-oxide synthase-2 linkage to focal adhesion kinase in neutrophils influences enzyme activity and beta2 integrin function. J Biol Chem.

[67] Hynes RO (2002). Integrins: bidirectional, allosteric signaling machines. Cell.

[68] Guan JL (1997). Role of focal adhesion kinase in integrin signaling. Int J Biochem Cell Biol.

[69] Cary LA, Guan JL (1999). Focal adhesion kinase in integrin-mediated signaling. Front Biosci.

[70] Kong D, Chen F, Sima NI (2015). Inhibition of focal adhesion kinase induces apoptosis in bladder cancer cells via Src and the phosphatidylinositol 3-kinase/Akt pathway. Exp Ther Med.

[71] Buhrmann C, Shayan P, Goel A, Shakibaei M (2017). Resveratrol regulates colorectal cancer cell invasion by modulation of focal adhesion molecules. Nutrients.

[72] Bragdon B, Moseychuk O, Saldanha S, King D, Julian J, Nohe A (2011). Bone morphogenetic proteins: a critical review. Cell Signal.

[73] Zhang L, Ye Y, Long X, Xiao P, Ren X, Yu J (2016). BMP signaling and its paradoxical effects in tumorigenesis and dissemination. Oncotarget.

[74] Cassar L, Nicholls C, Pinto AR, Chen R, Wang L, Li H (2017). TGF-beta receptor mediated telomerase inhibition, telomere shortening and breast cancer cell senescence. Protein Cell.

[75] Leinhauser I, Richter A, Lee M, Hofig I, Anastasov N, Fend F (2015). Oncogenic features of the bone morphogenic protein 7 (BMP7) in pheochromocytoma. Oncotarget.

[76] Fresno Vara JA, Casado E, de Castro J, Cejas P, Belda-Iniesta C, Gonzalez-Baron M (2004). PI3K/Akt signalling pathway and cancer. Cancer Treat Rev.

[77] Zeng YH, Zhou LY, Chen QZ, Li Y, Shao Y, Ren WY (2017). Resveratrol inactivates PI3K/Akt signaling through upregulating BMP7 in human colon cancer cells. Oncol Rep.

[78] Chen HJ, Hsu LS, Shia YT, Lin MW, Lin CM (2012). The beta-catenin/TCF complex as a novel target of resveratrol in the Wnt/beta-catenin signaling pathway. Biochem Pharmacol.

[79] Wolter F, Akoglu B, Clausnitzer A, Stein J (2001). Downregulation of the cyclin D1/Cdk4 complex occurs during resveratrol-induced cell cycle arrest in colon cancer cell lines. J Nutr.

[80] Suzuki R, Kuroda H, Komatsu H, Hosokawa Y, Kagami Y, Ogura M (1999). Selective usage of D-type cyclins in lymphoid malignancies. Leukemia.

[81] Carlson B, Lahusen T, Singh S, Loaiza-Perez A, Worland PJ, Pestell R (1999). Down-regulation of cyclin D1 by transcriptional repression in MCF-7 human breast carcinoma cells induced by flavopiridol. Cancer Res.

[82] Hsieh TC, Wu JM (1999). Differential effects on growth, cell cycle arrest, and induction of apoptosis by resveratrol in human prostate cancer cell lines. Exp Cell Res.

[83] Fontecave M, Lepoivre M, Elleingand E, Gerez C, Guittet O (1998). Resveratrol, a remarkable inhibitor of ribonucleotide reductase. FEBS Lett.

[84] Sengottuvelan M, Deeptha K, Nalini N (2009). Influence of dietary resveratrol on early and late molecular markers of 1,2-dimethylhydrazine-induced colon carcinogenesis. Nutrition.

[85] Gescher A, Steward WP, Brown K (2013). Resveratrol in the management of human cancer: how strong is the clinical evidence?. Ann N Y Acad Sci.

[86] Patel KR, Scott E, Brown VA, Gescher AJ, Steward WP, Brown K (2011). Clinical trials of resveratrol. Ann N Y Acad Sci.

[87] Chow HH, Garland LL, Hsu CH, Vining DR, Chew WM, Miller JA (2010). Resveratrol modulates drug- and carcinogen-metabolizing enzymes in a healthy volunteer study. Cancer Prev Res.

[88] Howells LM, Berry DP, Elliott PJ, Jacobson EW, Hoffmann E, Hegarty B (2011). Phase I randomized, double-blind pilot study of micronized resveratrol (SRT501) in patients with hepatic metastases-safety, pharmacokinetics, and pharmacodynamics. Cancer Prev Res.

[89] Liang L, Liu X, Wang Q, Cheng S, Zhang S, Zhang M (2013). Pharmacokinetics, tissue distribution and excretion study of resveratrol and its prodrug 3,5,4’-tri-O-acetylresveratrol in rats. Phytomedicine.

[90] Johnson JJ, Nihal M, Siddiqui IA, Scarlett CO, Bailey HH, Mukhtar H (2011). Enhancing the bioavailability of resveratrol by combining it with piperine. Mol Nutr Food Res.

[91] la Porte C, Voduc N, Zhang G, Seguin I, Tardiff D, Singhal N (2010). Steady-State pharmacokinetics and tolerability of trans-resveratrol 2000 mg twice daily with food, quercetin and alcohol (ethanol) in healthy human subjects. Clin Pharmacokinet.

[92] Smoliga JM, Blanchard O (2014). Enhancing the delivery of resveratrol in humans: if low bioavailability is the problem, what is the solution?. Molecules.

[93] Wang S, Su R, Nie S, Sun M, Zhang J, Wu D (2014). Application of nanotechnology in improving bioavailability and bioactivity of diet-derived phytochemicals. J Nutr Bioch.

[94] Paller CJ, Rudek MA, Zhou XC, Wagner WD, Hudson TS, Anders N (2015). A phase I study of muscadine grape skin extract in men with biochemically recurrent prostate cancer: safety, tolerability, and dose determination. Prostate.

[95] Kjaer TN, Ornstrup MJ, Poulsen MM, Jorgensen JO, Hougaard DM, Cohen AS (2015). Resveratrol reduces the levels of circulating androgen precursors but has no effect on, testosterone, dihydrotestosterone, PSA levels or prostate volume. A 4-month randomised trial in middle-aged men. Prostate.

[96] Jazirehi AR, Bonavida B (2004). Resveratrol modifies the expression of apoptotic regulatory proteins and sensitizes non-Hodgkin’s lymphoma and multiple myeloma cell lines to paclitaxel-induced apoptosis. Mol Cancer Ther.

[97] Popat R, Plesner T, Davies F, Cook G, Cook M, Elliott P (2013). A phase 2 study of SRT501 (resveratrol) with bortezomib for patients with relapsed and or refractory multiple myeloma. Br J Haematol.

[98] Sale S, Tunstall RG, Ruparelia KC, Potter GA, Steward WP, Gescher AJ (2005). Comparison of the effects of the chemopreventive agent resveratrol and its synthetic analog trans 3,4,5,4’-tetramethoxystilbene (DMU-212) on adenoma development in the Apc(Min +) mouse and cyclooxygenase-2 in human-derived colon cancer cells. Int J Cancer.

[99] Schneider Y, Vincent F, Duranton B, Badolo L, Gosse F, Bergmann C (2000). Anti-proliferative effect of resveratrol, a natural component of grapes and wine, on human colonic cancer cells. Cancer Lett.

[100] Nguyen AV, Martinez M, Stamos MJ, Moyer MP, Planutis K, Hope C (2009). Results of a phase I pilot clinical trial examining the effect of plant-derived resveratrol and grape powder on Wnt pathway target gene expression in colonic mucosa and colon cancer. Cancer Manag Res.

[101] Zhu W, Qin W, Zhang K, Rottinghaus GE, Chen YC, Kliethermes B (2012). Trans-resveratrol alters mammary promoter hypermethylation in women at increased risk for breast cancer. Nutr Cancer.

